# Malaria prevention in north-eastern Tanzania: patterns of expenditure and determinants of demand at the household level

**DOI:** 10.1186/1475-2875-8-95

**Published:** 2009-05-07

**Authors:** Brendan McElroy, Virginia Wiseman, Fred Matovu, William Mwengee

**Affiliations:** 1 Department of Economics, University College, Cork, Ireland; 2 Health Policy Unit & Gates Malaria Partnership, London School of Hygiene and Tropical Medicine, 50 Bedford Square, London, WCIB 3DP, UK; 3 Ministry of Health, Tanga Region, Tanzania

## Abstract

**Objective:**

This study aims to provide a better understanding of the amounts spent on different malaria prevention products and the determinants of these expenditures.

**Methods:**

1,601 households were interviewed about their expenditure on malaria mosquito nets in the past five years, net re-treatments in the past six months and other expenditures prevention in the past two weeks. Simple random sampling was used to select villages and streets while convenience sampling was used to select households. Expenditure was compared across bed nets, aerosols, coils, indoor spraying, using smoke, drinking herbs and cleaning outside environment.

**Findings:**

68% of households owned at least one bed net and 27% had treated their nets in the past six months. 29% were unable to afford a net. Every fortnight, households spent an average of US $0.18 on nets and their treatment, constituting about 47% of total prevention expenditure. Sprays, repellents and coils made up 50% of total fortnightly expenditure (US$0.21). Factors positively related to expenditure were household wealth, years of education of household head, household head being married and rainy season. Poor quality roads and living in a rural area had a negative impact on expenditure.

**Conclusion:**

Expenditure on bed nets and on alternative malaria prevention products was comparable. Poor households living in rural areas spend significantly less on all forms of malaria prevention compared to their richer counterparts. Breaking the cycle between malaria and poverty is one of the biggest challenges facing malaria control programmes in Africa.

## Background

The provision of insecticide-treated nets (ITNs) in malarious regions is widely accepted as an essential public health service [1,2]. One of the key reasons cited for this is that when used properly, intact ITNs provide almost complete protection from mosquito bites [3]. Studies have also demonstrated the efficacy of ITNs with an overall reduction in all-cause mortality of 17% [4]. The cost-effectiveness of ITNs relative to other forms of malaria prevention and treatment has also been widely demonstrated [5-10].

However, many households also use other methods of prevention such as indoor spraying, aerosols, coils and smoking of herbs [11,12]. According to some experts in the field, some of these products are likely to give partial protection [13,14]. However, the scientific evidence needed to support these claims is sparse. Some studies show that coils can have a protective effect against mosquito biting but this varies according to the ingredients and the conditions under which they are used [15-21].

Little is known about the level of household expenditure on these non-net products and practices. The evidence that does exist suggests that households, especially those in urban settings and in rural cash economies, spend considerable amounts of money on commercial repellents such as mosquito coils [22-24]. Households in poorer rural areas tend to choose traditional methods such as burning local herbs [25-27]. In addition, the poor spend a larger proportion of household income on these prevention activities than the rich [28,29].

Relatively more is known about the factors influencing the demand for bed nets. For example, a growing number of studies have reported significant financial barriers to their use [24-26,29-33] A range of demographic variables including gender, age, education, household size and ethnicity, have all been shown to influence bed net use [24,34-36]. Community infrastructure is also important [24].

This study seeks to identify how much households spend on a comprehensive range of malaria prevention products. Expenditure is also analysed by socio-economic status. This involved quantifying the size of differentials in expenditure across socio-economic groups. Finally, the factors affecting expenditure decisions are examined, to better inform malaria control programmes.

## Methods

### Study site

Data collection was carried out between September 2003 and February 2005 in rural and urban areas of Tanga district. The climate is monsoonal with short *vuli *rains from October–December and long *masika *rains from March–May. There are four dominant ethnic groups: the Digo, Bondei, Wazigua and Sambaa, with over 20 other smaller ethnic groups. The main economic activities in rural areas are subsistence farming and fishing with limited cash-cropping (cashews and palm trees). The majority of households in the urban centres are engaged in trade, varying from wholesale stores to petty trade in small markets and along streets. A few others work on sisal plantations.

Drug stores and pharmacies frequently stock bed nets and other malaria prevention products such as repellents and sprays. Bed nets are also available in some general retail shops. Bed nets are usually packed together with insecticide. At the time of the study, the average price for a bed net was about US $3 and the insecticide (solution or tablet) about US $0.20.

### Sample

A sample of 1,601 rural and urban households was selected using a combination of sampling methods. The primary sampling unit was the street/sub-village. Cluster sampling occurred at ward and village level, simple random sampling at street and sub-village level. A ward is an administratively demarcated area below the district level, which may comprise 3–5 villages (rural) or 6–14 streets (urban), and a population of between 2500 to 26000 people (about 600 to 5500 households) (Republic of Tanzania, 2002).

First, eight wards were randomly selected in the urban areas and five in the rural areas, representing 80% and 83% of the wards in each respective area. For each of the urban wards sampled, four streets were randomly selected. From the five rural wards, eight villages were randomly selected, and five sub-villages were randomly selected from each village. In total, 32 streets (22% of all streets) and 41 sub-villages (32% of all sub-villages) were selected. Each street or sub-village was given equal weight so the sampling percentage of streets per ward varied from 29% to 67% and the sampling percentage of sub-village per village varied from 56% to 83%. A sub-village had between 20 and 50 households, and a street between 100 and 150 households. For logistical reasons, convenience sampling was used to select households from within sub-villages and streets. Chosen households had to be at least 4 households apart, and approximately 10% of the households in a sub-village or street were interviewed. The percentage of the total population surveyed was 2.4% in urban areas and 8.3% in rural areas respectively.

### Data collection

Data were collected by four field workers, all of whom had completed advanced secondary school and were trained in data collection skills prior to the survey. The questionnaire was administered in Swahili to the household head or their spouse. If the household head was not available, then the family was asked to nominate the next most appropriate person.

Three different time frames were used to assess expenditure on malaria prevention. First, respondents were asked how much they spent on the bed nets they currently owned. Second, they were asked about how much they spent on the treatment and repair of bed nets over the previous six months. Third, they were asked about expenditures on other forms of malaria prevention over the previous two weeks including expenditure on coils, indoor sprays, aerosols, repellents, herbs, cleaning surrounding environment and clearing vegetation; and any other forms of prevention. It was assumed that bed nets last for five years [9,24,37,38] and that insecticide on bed nets lasts for six months [39]. To make comparisons across the three time frames, expenditure on bed nets was divided by 130 (26 fortnightly periods per year by five years) and expenditure on the treatment and repair of bed nets over six months was divided by 13.

Respondents were also asked about demographic and socio-economic characteristics of the household. The various approaches to survey design for developing countries, including pitfalls to be avoided during data collection, that are discussed comprehensively in [40] guided this study. While the questionnaire was designed specifically for this study, the overall structure and some questions on treatment seeking and expenditure were similar to those used in the World Bank Living Standards Measurement Surveys, as described in [40].

The pre-coded prevention strategies presented in the household interviews were based on a series of seven focus group discussions. Each group involved an average of 12 participants who were purposively sampled from each of the dominant ethnic and gender groups. During these discussions, community members were asked to describe the main products or practices that could be used to prevent malaria. Survey instruments were translated into Swahili and piloted on men and women who had varying levels of education and were from both rural and urban areas. Questionnaires were double entered using Access 7.0. All estimates are reported in US dollars, based on a conversion rate of: US$1 = 1,302 TSH.

Interviews were also held with 47 community spokespersons between January and March 2004. Respondents were purposively sampled and interviewed about community level factors that were likely to influence the level of expenditure on malaria prevention such as the quality of roads, access to clean water, access to formal education and the presence of markets and shops.

Clearance for this research was obtained from the National Institute for Medical Research (NIMR) of the Republic of Tanzania, the Regional Medical Office in Tanga, and the London School of Hygiene and Tropical Medicine. Written informed consent was obtained from all participants. Community leaders were briefed about the study objectives and design.

### Analysis

Analysis was performed in STATA 8. Sampling weights were computed to adjust for the different probability of being selected in the urban and rural areas.

Access to material resources was measured using an index of household wealth. This approach is becoming increasingly popular in health services research in developing countries [24,41-44]. It uses the first component from principal components analysis to assign weights to each asset. These weights maximize the variance in the list of assets. Consequently, assets that vary considerably between households get a large weight and those that vary little get a small weight. More formally, given an asset vector *x *= (*x*_1_, *x*_2_,..., *x*_*p*_,)', the first principal component of the observations, *y*, is the linear combination ,

whereby sample variance is maximized, subject to the restriction that *a*'*a *= 1. In this instance *a *is the vector of coefficients, and  and *s*_*k *_are the mean and standard deviation of variable *x*_*k*_. The wealth index of household *i *with assets *x*_*i *_is , where  is the vector of standardized variables above. The wealth index has zero mean and variance *λ*, where *λ *is the largest eigenvalue of the correlation matrix of the asset vector *x *[43]. Data on the quantities owned of each of fourteen assets were collected, namely, bicycle, cart, bed, motorbike, radio, TV, tin roof, watch, foam mattress, cattle, donkeys, goats, horses, sheep, chickens.

Since socio-economic status is a broader concept than simply access to material resources, an index of socio-economic status was also generated. This comprised of the 14 assets used for the wealth index, years of education of adults in the household and occupation of the household head.

The following list of variables is posited as determinants of household prevention expenditure: household demographic characteristics; socio-demographic characteristics of the head of household; household socio-economic characteristics; community effects and seasonal effects.

The determinants of prevention expenditure are analysed using a generalized linear model (GLM) [45]. Expenditure data of the type analysed here are characterized by non-negativity, a spike at zero (referring to non-purchasers of prevention products) and a positive skew (referring to people who bought a considerable amount over the two weeks prior to the survey). As such, GLMs offer a much more appropriate regression framework than ordinary least squares (see [46] for a review). The GLM specification used here has a logarithmic link function and a gamma variance function. This specification is typical for health expenditure functions [46,47].

## Results

Table 1 shows individual and household level characteristics of the study sample. The average number of bed nets per household was 1.67 while the average number of ITNs (i.e. bed nets that had been treated with insecticide in the last six months) was 0.77. The average household size was 5.41, which has been broken down into eight age groups each of approximately 0.6 persons each. The average household head was aged 44 years; 79% of them were married and they had an average number of years of education of 6.54. The average number of years of education of all household adults was just lower at 6.29. The four main ethnic groups of Sambaa, Digo, Bondei and Pare constituted approximately 60% of the total sample. The wealth index had zero mean (by construction) and varied from a low of -2.39 to a high of 17.80. The index of socio-economic status varied from -3.35 to 16.50. Approximately two-thirds of household heads were businessmen or in agriculture-related work. Fifty-four percent of households lived in rural areas. Seven percent of communities had roads capable of carrying vehicles. Meanwhile, the road to approximately 72% of households was impassable at certain times of the year. Forty-two percent of households were in communities served by public transport.

**Table 1:** Characteristics of respondents and their household

Variable	Mean^1^	Std. Dev.	Min	Max
*Prevention Measures*				
Number of bed nets per household	1.67	1.66	0.00	9.00
Number of ITNs per household	0.77	1.49	0.00	8.00
Unit value of bed net	3,840.89	1,189.56	0.00	10,250
*Household Demographics*				
No. in household aged 0–4 years	0.78	0.79	0.00	5.00
No. in household aged 5–9 years	0.80	0.86	0.00	5.00
No. in household aged 10–14 years	0.78	0.90	0.00	5.00
No. in household aged 15–19 years	0.57	0.82	0.00	5.00
No. in household aged 20–29 years	0.86	0.92	0.00	7.00
No. in household aged 30–39 years	0.67	0.73	0.00	6.00
No. in household aged 40–54 years	0.55	0.70	0.00	4.00
No. in household aged 55 and over	0.41	0.68	0.00	4.00
No. in household	5.41	2.38	1.00	17.00
*Household Socio-economics*				
Index of household wealth	0.00	1.95	-2.39	17.80
Index of socioeconomic status	0.00	2.10	-3.35	16.80
*Socio-demographics of head*				
Age of household head	43.62	14.20	0.00	92.00
Head is married = 1	0.79	0.41	-	-
Yrs of education of head	6.54	3.15	0.00	16.00
Head occupation is business	0.23	0.43	-	-
Head occupation is agriculture	0.39	0.49	-	-
Head occupation is civil servant	0.07	0.26	-	-
Head occupation is other	0.26	0.44	-	-
*Ethnicity*				
Head is Sambaa	0.13	0.33	-	-
Head is Digo	0.36	0.48	-	-
Head is Bondei	0.06	0.23	-	-
Head is Pare	0.05	0.21	-	-
Head is from other ethnic group	0.34	0.47	-	-
*Community variables*				
Rural household	0.54	0.50	-	-
On public transport route	0.42	0.49	-	-
Road capable of carrying vehicles	0.07	0.25	-	-
Community's road is impassable during year	0.72	0.44	-	-

Note 1: Mean reported for continuous or integer variables. Proportion of the total sample that is '1' reported for dummy variables.

Note 2: Refers to treated and untreated nets

Table 2 shows that over the past two weeks, households spent an average of US$0.41 (533.55 TSH) on malaria prevention. Average expenditure on nets was US $0.18 over a two-week period. Expenditure on the treatment and repair of nets was more modest, at US $0.03. Nets (including treatment and repair) made up 47% of fortnightly expenditure on prevention, while sprays, repellents and coils made up 50%. Few people engaged in the remaining prevention measures including aerosols, cleaning the environment and 'other' prevention. Approximately 8% of respondents invested in household spraying but it constituted 25% of total expenditure, indicating that it is a relatively expensive activity. By contrast, coils are used by approximately 15% of the sample but represent only 11% of total expenditure.

**Table 2:** Expenditure and use of Malaria Prevention Measures

**Prevention measure**	**Sample mean (US$)**	**% of respondents engaging in activity**	**Mean expenditure over 2 weeks (US$)**	**% of total expenditure over 2 weeks**
Bed nets	6.96	0.68	0.18	43
Treatment of bed nets	0.74	0.27	0.02	4
Repair of bed nets	0.07	0.26	< 0.01	0
Aerosols	0.01	0.01	0.01	1
Coils	0.04	0.15	0.04	11
Clean environment	< 0.01	0.13	< 0.01	1
Clear vegetation	-	0.01	-	-
Eat/drink herbs	< 0.01	0.01	< 0.01	0
Spray	0.10	0.08	0.10	25
Repellents	0.06	0.05	0.06	14
Use smoke	-	0.02	-	-
Other	< 0.01	0.05	< 0.00	1

Total			0.41	100

1: Reference time frames are five years for nets; six months for treatment and repair of nets; and two weeks for the remaining prevention measures.

Of the 33% (n = 528) of households who did not own a bed net (treated or untreated), approximately 87% (n = 461) said it was because they could not afford one. Table 3 reveals that they spent less than US $0.09 on other forms of prevention per fortnight.

**Table 3:** Fortnightly expenditure on prevention by respondents who state they cannot afford a net

**Prevention measure (n = 461 or 29%)**	**Average fortnightly expenditure (US$)**
Aerosols	0.00
Coils	0.07
Clean environment	0.00
Clear vegetation	0.00
Eat/drink herbs	0.00
Spray	0.01
Repellents	0.01
Use smoke	0.00
Other	< 0.01

Total	0.09

**Figure 1:**
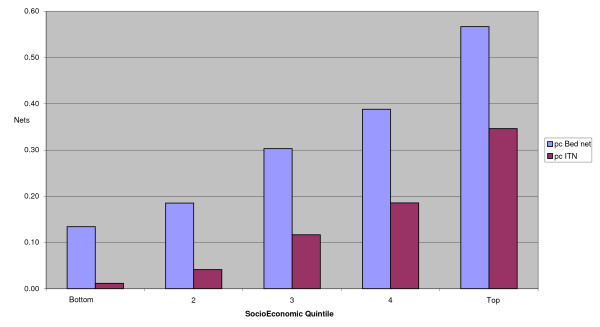
**Number of bed nets and ITNs per capita by SES**.

**Figure 2:**
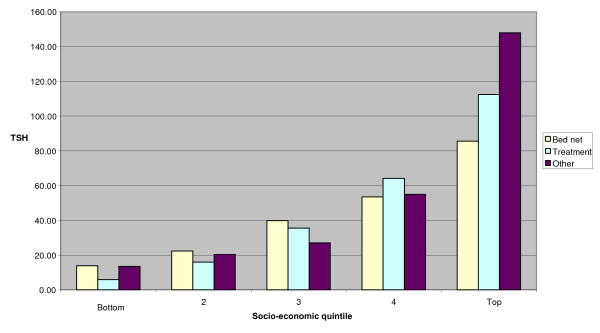
**Per capita expenditure on prevention by SES**.

To further examine affordability, Figures 1 and 2 examine the relationship between socioeconomic status and malaria prevention more closely. Figure 1 shows that bed net ownership significantly increased with socioeconomic status. Households in the lowest quintile owned an average of around one bed net (0.13 per capita) compared to those in the highest quintile who owned between five and six nets (0.57 per capita), or 4.2 times more per capita. The difference is even starker for ITNs. Households in the lowest quintile owned an average of 0.01 ITNs per capita compared to those in the highest quintile who owned an average of 0.35 ITNs per capita, or 29.5 times more per capita.

Figure 2 reveals that expenditure on all forms of malaria prevention increased with socioeconomic status. The top socioeconomic quintile spent 6.2 times more on bed nets and 19.2 times more on treating nets than the bottom one. In addition, the top quintile spent more on 'other' forms of prevention than all the other quintiles put together. The bottom four quintiles spend approximately 50% of total expenditure on bed nets, whereas the top quintile spends only 35%, further highlighting that quintile's disproportionate spend on 'other' forms of prevention.

Table 4 exhibits the determinants of prevention expenditure. The sample size is reduced to 1498 for Table 4 because no community variables were available for 103 respondents. The R^2 ^of 43.5% indicates a high degree of explanatory power. Coefficients are expressed in logarithms. Significance testing is based on heteroscedasticity robust standard errors. A less restricted model that included the ethnicity variables reported in Table 1 was tested and rejected using a Wald test.

**Table 4:** Determinants of expenditure on prevention

**Variable**	**Coefficient**
*Household Demographics*	
No. in household aged 0–4 years	0.010
No. in household aged 5–9 years	0.116**
No. in household aged 10–14 years	0.026
No. in household aged 15–19 years	0.112**
No. in household aged 20–29 years	0.120**
No. in household aged 30–39 years	0.076
No. in household aged 40–54 years	0.139
No. in household aged 55 and over	-0.042
*Household Socio-economics*	
Wealth index	0.267**
Square of wealth index	-0.014**
*Socio-demographics of head*	
Age of head	0.003
Head is married	-0.195**
Years of education of head	0.118**
Square of years of education of head	-0.002
Head occupation is agriculture	-0.170*
Head occupation is civil service	-0.192*
Head occupation is other	-0.151*
*Community variables*	
Rural household	-1.041**
On public transport route	-0.476**
Poor quality roads	-0.181
Community's road is impassable during year	-0.257**
*Seasonal variables*	
Vuli1	0.319**
Vuli2	-0.045
Masika	-0.079
Constant	5.958**
N	1498
Pseudo-R^2^	0.435

* Significant at 10% level

** Significant at 5% level

Three of the age groups under 30 (age 5 – 9, age 15 – 19 and age 20 – 29) were positive and highly significant. Although pre-school children are more vulnerable to the damaging effects of malaria infection, the age 0 – 4 group was insignificant.

Wealthier households spent more on malaria prevention but this declined with increased levels of wealth, as indicated by the negative sign on the 'wealth squared' term. Years of education of the household head had a strong positive effect, while being married had a negative effect.

Compared to heads who were businessmen (including retailers), which was the reference occupational group, farmers, civil servants and 'others' spent less, at the 10% level of significance. Overall, community variables had a considerable negative effect, with rural residence having the highest negative coefficient in the model. These highlight the role of access to markets in determining prevention expenditure.

Finally, a seasonal effect was detected for the first vuli rains (October to December). An alternative specification for seasonality based on a 'monsoon' dummy variable consisting of all three seasons was insignificant.

## Discussion

This study revealed that households spend an average of approximately $0.41 per fortnight on malaria prevention with bed nets making up almost half of this amount. Repellents, sprays, and coils made up the remaining half. In comparison, a random sub sample of households (n = 308) taking part in this study reported that they spent US$4.35 on education and about US$4.82 on health care per fortnight. Other studies show a greater proportion of household income spent on repellents, coils and aerosols in Burkina Faso [22], Ghana [12,28,36], Tanzania [48] and The Gambia [25].

Looking more closely at these results, it also becomes clear that the poorest segments of society are spending significantly less on all forms of malaria prevention than their richer counterparts. For example, it was demonstrated that the top socioeconomic quintile spent 6.2 times more on bed nets and 19.2 times more on treating nets than the bottom one. Affordability was cited as the main reason for not owning a bed net. This is consistent with a number of studies that show that the use of preventive measures, particularly ITNs is strongly and positively correlated with income and socioeconomic status [25,33,48-50]. Interestingly, poor households that spent a meagre amount on malaria prevention, tended to choose nets over other products such as coils and aerosols. This is in direct contrast to other studies that have shown that even the poorest households will often spend more per month on coils, sprays and repellents, than the equivalent actualized monthly cost of owning an ITN [25,51,52].

This study also revealed that the poorest quintile owned far fewer treated nets than untreated relative to the richest quintile. Households in the lowest quintile owned an average of 0.01 ITNs per capita compared to those in the highest quintile who owned an average of 0.35 ITNs per capita, or 29.5 times more per capita. These data indicate that increasing the use of ITNs (especially amongst the poor) by providing insecticide treatment for any untreated nets already in houses may be an effective way of increasing coverage amongst poorer groups. Much depends however on the quality of the nets being re-treated [53].

This study also indicates that in terms of household expenditure on malaria prevention, households with children under 5 do not spend more on nets than other households. Prevention expenditure was shown to be positive and highly significant across all age groups under 30 except for children under five. This may reflect the work of charitable organizations such as Tanga Rotary Club that occasionally distribute small quantities of free nets. During the course of this study, Population Services International (PSI) was also involved in net promotion through public health campaigns, promoting the net manufacturers of four leading brands, and encouraging bundling nets together with insecticide (Jane Miller personal communication). However despite these positive initiatives, a recent study of bed net utilization in Tanga shows that under-five utilization of ITNs remains disturbingly low with only 10% of rural under fives and 47% of urban under fives using a treated net [54]. This falls well short of the RBM target of 80%.

This study also implies that the demand for malaria prevention can be significantly increased if aspects of public infrastructure are improved especially in rural areas. Rural households spent significantly less on malaria prevention compared with households in urban areas and roads to these communities were more likely to flood during the wet season. A study from The Gambia also found that poor quality roads played a key role in dampening the demand for bed nets [25]. While there has been a tendency in previous studies to focus on individual or household level determinants of demand, there is growing recognition that the task of malaria control cannot be left entirely to health services.

Two methodological issues should be borne in mind. First, participants in this study were specifically asked about activities for preventing malaria as opposed to mosquito avoidance practices. While households often have a good understanding of the relationship between mosquitoes and malaria [11], the main reason for using products such as bed nets is to prevent nuisance mosquito bites [55]. There is, therefore, the danger that not all relevant areas of expenditure on malaria prevention are captured. The high level of overlap between activities identified in this study and those reported in studies of mosquito protection in Africa [11] suggest that this was not a major problem.

Secondly, there is much debate over the use of wealth indexes to measure access to material resources. Collecting information on asset ownership is generally quicker and less vulnerable to problems of recall bias and mis-measurement of asset ownership compared to income and consumption surveys in developing countries [43]. Asset-based wealth measures however often overlook the fact that 'poor' individuals often live in relatively wealthy households [56]. Moreover, wealth indexes are commonly computed over pooled data for rural and urban households to allow for direct comparability of households in different areas. Some assets such as iron-roofed houses (an asset used in this study) tend to vary more in the rural areas than in the urban. This implies that the weight attached to an iron-roofed house would be higher in a rural-specific index, allowing greater discrimination between rural households. It is possible that the significant urban/rural differentials reported in this study may actually be picking up wealth differences not accurately captured by the asset index. To investigate this issue, separate urban and rural indices were included in an unreported model and the interpretation of almost all other variables was similar, the exception being occupation, where agriculture became highly significant and the other two occupational groups became insignificantly different from businessmen.

## Conclusion

Unlike most previous studies, this study does not show that poor households are investing a wide variety of prevention products that absorb a significant amount of their income. Moreover, what little money is being spent on malaria prevention is often directed towards bed nets. Nonetheless, cost continues to prevent the majority of households from purchasing a net. To further stimulate demand for ITNs, especially amongst the rural poor, financial barriers must be addressed. The Tanzanian government is attempting to do just this through a programme of vouchers for pregnant women provided at ANCs, which cover about 75% of the regular market price of an ITN. Vouchers can be exchanged for an untreated net bundled with insecticide at pre-determined retailers (drug shops, pharmacies and retail shops) or government health care facilities upon payment of the remaining balance [57]. The Global Fund is also supporting a national campaign involving the free distribution of long-lasting insecticide-treated bed nets to all children under five years of age, that is due to commence this year in Tanzania. The government might also consider providing insecticide treatment for any untreated nets already in houses as an effective way of increasing coverage amongst poorer groups. Improving the quality of roads in rural areas is another effective means of stimulating demand for malaria prevention products.

## Competing interests

The authors declare that they have no competing interests.

## Authors' contributions

VW and WM were Principal Investigators. VW & BMcE led the drafting of the manuscript. BMcE performed quantitative analysis. FM managed the data collection process. All authors contributed to study design and interpretation of results. All authors read and approved the final manuscript.
